# Persistent Infection of a Canine Histiocytic Sarcoma Cell Line with Attenuated Canine Distemper Virus Expressing Vasostatin or Granulocyte-Macrophage Colony-Stimulating Factor

**DOI:** 10.3390/ijms23116156

**Published:** 2022-05-31

**Authors:** Katarzyna Marek, Federico Armando, Vanessa Maria Nippold, Karl Rohn, Philippe Plattet, Graham Brogden, Gisa Gerold, Wolfgang Baumgärtner, Christina Puff

**Affiliations:** 1 Department of Pathology, University of Veterinary Medicine Hannover, 30559 Hannover, Germany; katarzyna.marek@tiho-hannover.de (K.M.); federico.armando@tiho-hannover.de (F.A.); pfankuche@laboklin.com (V.M.N.); christina.puff@tiho-hannover.de (C.P.); 2 Center for Systems Neuroscience, University of Veterinary Medicine Hannover, 30559 Hannover, Germany; 3 Institute for Biometry, Epidemiology and Information Processing, University of Veterinary Medicine Hannover, 30559 Hannover, Germany; karl.rohn@tiho-hannover.de; 4 Division of Experimental Clinical Research, Vetsuisse University Bern, 3012 Bern, Switzerland; philippe.plattet@vetsuisse.unibe.ch; 5 Department of Biochemistry, University of Veterinary Medicine Hannover, 30559 Hannover, Germany; graham.brogden@tiho-hannover.de (G.B.); gisa.gerold@tiho-hannover.de (G.G.); 6 Research Center for Emerging Infections and Zoonoses (RIZ), University of Veterinary Medicine Hannover, 30559 Hannover, Germany; 7 Institute for Experimental Virology, TWINCORE, Centre for Experimental and Clinical Infection Research, a Joint Venture between the Medical School Hannover and the Helmholtz Centre for Infection Research, 30625 Hannover, Germany; 8 Wallenberg Centre for Molecular Medicine (WCMM), Umeå University, 901 87 Umeå, Sweden; 9 Department of Clinical Microbiology, Virology, Umeå University, 901 87 Umeå, Sweden

**Keywords:** canine distemper virus, canine histiocytic sarcoma, CDV-Onderstepoort, DH82, genetically engineered viruses, GM-CSF, mNeonGreen, vasostatin, viral oncolysis

## Abstract

Canine histiocytic sarcoma (HS) represents a neoplasia with poor prognosis. Due to the high metastatic rate of HS, there is urgency to improve treatment options and to prevent tumor metastases. Canine distemper virus (CDV) is a single-stranded negative-sense RNA (ssRNA (-)) virus with potentially oncolytic properties. Moreover, vasostatin and granulocyte-macrophage colony-stimulating factor (GM-CSF) are attractive molecules in cancer therapy research because of their anti-angiogenetic properties and potential modulation of the tumor microenvironment. In the present study, an *in vitro* characterization of two genetically engineered viruses based on the CDV strain Onderstepoort (CDV-Ond), CDV-Ond^neon-vasostatin^ and CDV-Ond^neon-GM-CSF^ was performed. Canine histiocytic sarcoma cells (DH82 cells) were persistently infected with CDV-Ond, CDV-Ond^neon^, CDV-Ond^neon-vasostatin^ and CDV-Ond^neon-GM-CSF^ and characterized on a molecular and protein level regarding their vasostatin and GM-CSF production. Interestingly, DH82 cells persistently infected with CDV-Ond^neon-vasostatin^ showed a significantly increased number of vasostatin mRNA transcripts. Similarly, DH82 cells persistently infected with CDV-Ond^neon-GM-CSF^ displayed an increased number of GM-CSF mRNA transcripts mirrored on the protein level as confirmed by immunofluorescence and Western blot. In summary, modified CDV-Ond strains expressed GM-CSF and vasostatin, rendering them promising candidates for the improvement of oncolytic virotherapies, which should be further detailed in future *in vivo* studies.

## 1. Introduction

Cancer is one of the leading causes of death in dogs [1] and canine histiocytic sarcoma (HS) represents an aggressive neoplasm with a poor prognosis [2]. Traditional treatment options often possess poor efficacy and can lead to considerable side effects [3]. Therefore, viral oncolysis is a novel, interesting approach as an alternative therapy. 

Viral oncolysis is based on the premise that virus infection, amplification and spread within a tumor leads to its eradication [4]. In general, the mechanisms of viral oncolysis comprise primary (e.g., direct induced apoptosis/cell lysis) and secondary effects [5]. The latter include a wide range of events including modulation of the anti-tumoral immune response, targeting matrix metalloproteinases (MMPs) and their inhibitors (TIMPs), and the inhibition of tumor-angiogenesis that may lead to tumor cell death [5].

Due to its close relationship to the measles virus, which has already been tested as an oncolytic agent in clinical studies in human medicine [6], canine distemper virus (CDV) might represent a promising candidate in veterinary medicine. Interestingly, CDV leads to apoptosis of canine neoplastic B and T lymphocytes [7] and human cervical carcinoma cells (HeLa cells) [8], which could broaden the spectrum of tumors which can be treated with the vaccine strains of CDV. So far, there are no data regarding modified CDV-Ond strains or the susceptibility of cells to these strains. In addition, previous studies have shown that an infection of canine histiocytic sarcoma cells (DH82 cells) with the CDV vaccine strain Onderstepoort (CDV-Ond) leads to changes in MMP expression *in vitro* [9] and in murine xenotransplantation studies [10]. Furthermore, an infection with CDV-Ond results in decreased tumor cell motility and invasiveness, reduced angiogenic gene expression, and a dysregulated HIF-1α downstream pathway *in vitro* [11,12,13]. Similarly, a xenotransplantation of persistently CDV-Ond-infected DH82 cells resulted in a complete, spontaneous regression of the neoplasms [14] and an acute infection of xentrotransplanted canine histiocytic sarcoma cells was associated with reduced tumor growth [15].

The inhibition of tumor angiogenesis is one of the most important starting points for anti-tumor therapies, as the continuous growth of tumors is dependent on a sufficient blood supply [16]. Vasostatin, the N-terminal domain of calreticulin, is a potent inhibitor of angiogenesis, which specifically targets proliferating endothelial cells, leading to the inhibition of cell proliferation [17]. Moreover, vasostatin expression is associated with a diminished growth rate and the metastasis of lung carcinoma in murine xenografts [18]. Furthermore, an inhibition of angiogenesis and the reduced growth of human colon carcinoma and Burkitt lymphoma xenografts has been reported *in vivo* [17]. Interestingly, the infection of heterotopic murine pancreatic carcinoma transplants with a recombinant adenovirus strain expressing vasostatin leads to growth retardation and decreased microvessel density [19]. 

The modulation of the tumor microenvironment represents another promising approach for anti-tumor therapy. Classically activated macrophages (M1) are known to be pro-inflammatory and anti-tumoral, while alternatively activated macrophages (M2) behave contrarily [20,21]. The increased presence of tumor-associated macrophages (TAMs) is associated with poor prognosis in many types of cancer [22], since they can promote secretion of pro-angiogenetic factors and promote tumor growth [20,23]. Due to this fact, the targeted polarization of TAMs in M1 macrophages may be a chance to prevent or revert an M2 phenotype within the neoplastic tissue. Moreover, it has been reported in humans that the polarization of M2 into M1 macrophages depends mainly on the presence of granulocyte and macrophage colony-stimulating factor (GM-CSF) [24,25].

Nowadays, genetic engineering can further enhance the anti-tumoral properties of viruses [26]. Indeed, the insertion of GM-CSF is one of the strategies already applied in other oncolytic viruses such as vaccinia virus, adenovirus, herpes simplex virus (T-VEC, Imlygic™), Newcastle disease virus, and measles virus [26,27]. The infection of human lymphoid tumor cells with measles virus expressing GM-CSF in murine xenografts leads to a markedly slower tumor progression compared to neoplasms infected with measles virus alone. This effect is attributed to the modulation of the tumor microenvironment [27].

Based on these previous findings and taking into consideration the results obtained in the canine histiocytic sarcoma model using DH82 cells, CDV-Ond represents a very promising candidate for oncolytic virotherapy. Therefore, CDV-Ond strains were engineered in order to express vasostatin or GM-CSF in infected cells to potentiate the therapeutic effect. The aim of this study was to characterize DH82 cells persistently infected with engineered CDV-Ond strains expressing either vasostatin (CDV-Ond^neon-vasostatin^) or GM-CSF (CDV-Ond^neon-GM-CSF^). 

## 2. Results

### 2.1. DH82 Cell Growth Rate and Virus Replication Ability Are Independent of the Virus Strain 

The current experiments were performed with early passages (passage 4 to 6) of all persistently infected cell lines.

**Figure 1:**
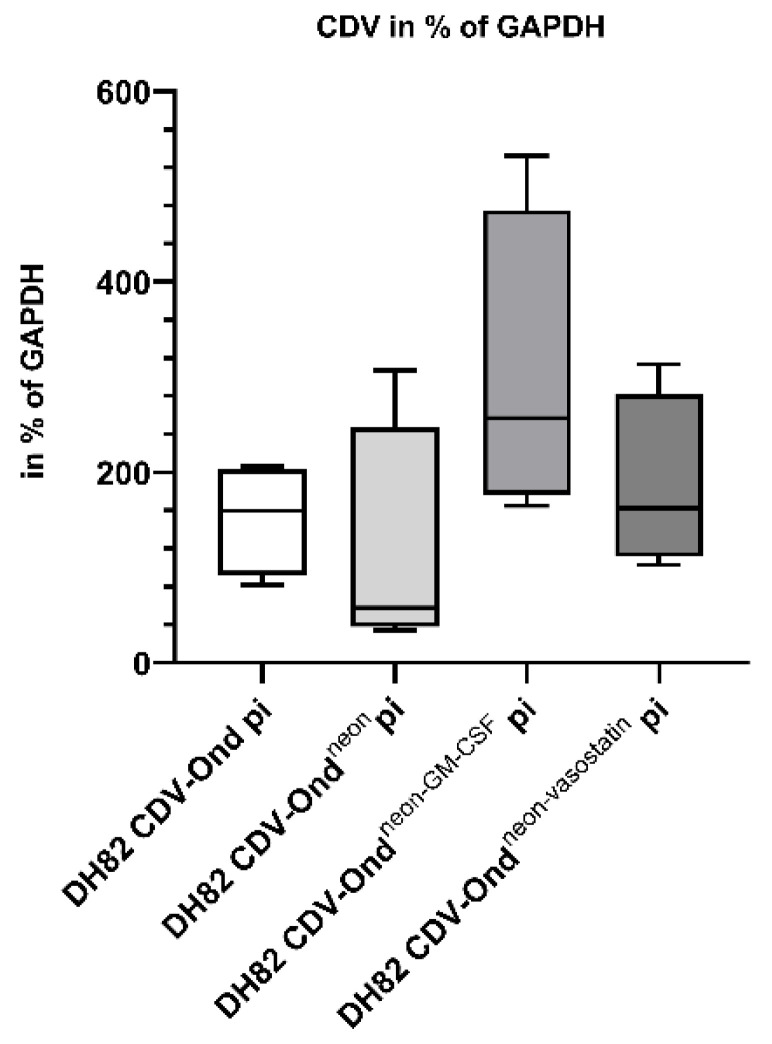
Results of the qPCR revealed no statistically significant differences between the different persistently infected cell lines (Mann–Whitney-U test; *p* > 0.05). Box plots represent minimum, first quartile, median, third quartile and maximum.

DH82 cells persistently infected with CDV-Ond^neon-GM-CSF^ and CDV-Ond^neon-vasostatin^ were compared to non-infected DH82 cells and DH82 cells persistently infected with CDV-Ond and CDV-Ond^neon^, respectively. The infection status of the aforementioned persistently infected cultures was assessed by immunofluorescence (IF) staining and reverse transcription quantitative PCR (RT-qPCR) for CDV-nucleoprotein (CDV-NP). CDV-NP was detected in more than 95% of the cells in infected cultures regardless of the strain, whereas non-infected controls were negative (Table 1, Supplementary Figure S1). Moreover, there were no significant differences between the number of CDV mRNA transcripts in persistently infected DH82 cell lines independent of the virus strain (*p* > 0.05), as shown in Figure 1. Non-infected DH82 cells served as controls and did not exhibit a CDV titer or CDV mRNA transcripts.

**Figure 2:**
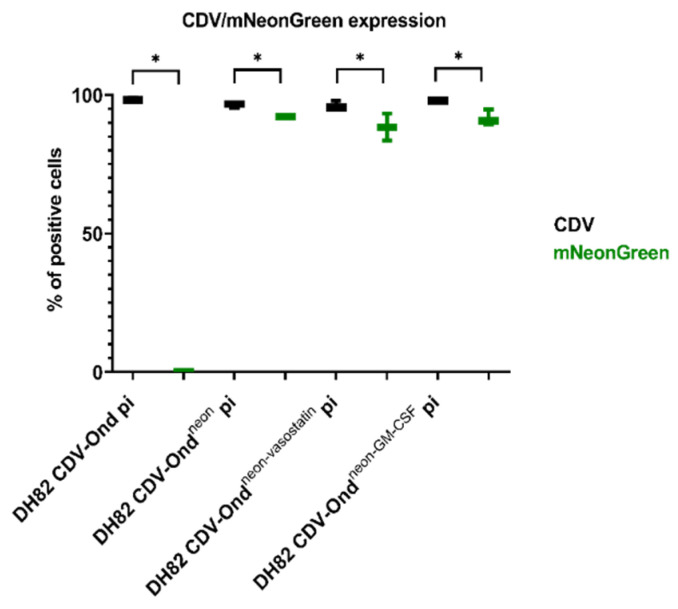
Statistical analysis revealed significant differences between the number of mNeonGreen-expressing and CDV nucleoprotein-immunopositive DH82 cells in cultures persistently infected with CDV-Ond, CDV-Ond^neon^, CDV-Ond^neon-GM-CSF^ and CDV-Ond^neon-vasostatin^ (Wilcoxon signed-rank test; *p* ≤ 0.05). Box plots represent minimum, median and maximum. Significant differences are labeled by asterisks * = *p* ≤ 0.05.

Furthermore, the percentage of mNeonGreen-positive cells was determined in all cultures. All investigated cultures infected with CDV-Ond engineered to express mNeonGreen (DH82 CDV-Ond^neon^ pi, CDV-Ond^neon-GM-CSF^ pi, CDV-Ond^neon-vasostatin^ pi) displayed more than 80% mNeonGreen-positive cells, whereas non-infected cultures and cells infected with DH82 CDV-Ond were negative (Table 2, Supplementary Figure S2). Statistical analysis revealed significant differences between the number of mNeonGreen-expressing and CDV nucleoprotein-immunopositive DH82 cells in cultures persistently infected with CDV-Ond, CDV-Ond^neon^, CDV-Ond^neon-GM-CSF^ and CDV-Ond^neon-vasostatin^ (Figure 2). The cumulative population doubling assay revealed a similar growth rate without statistically significant differences in all cultures independent from the virus strain (Supplementary Figure S3).

**Figure 3:**
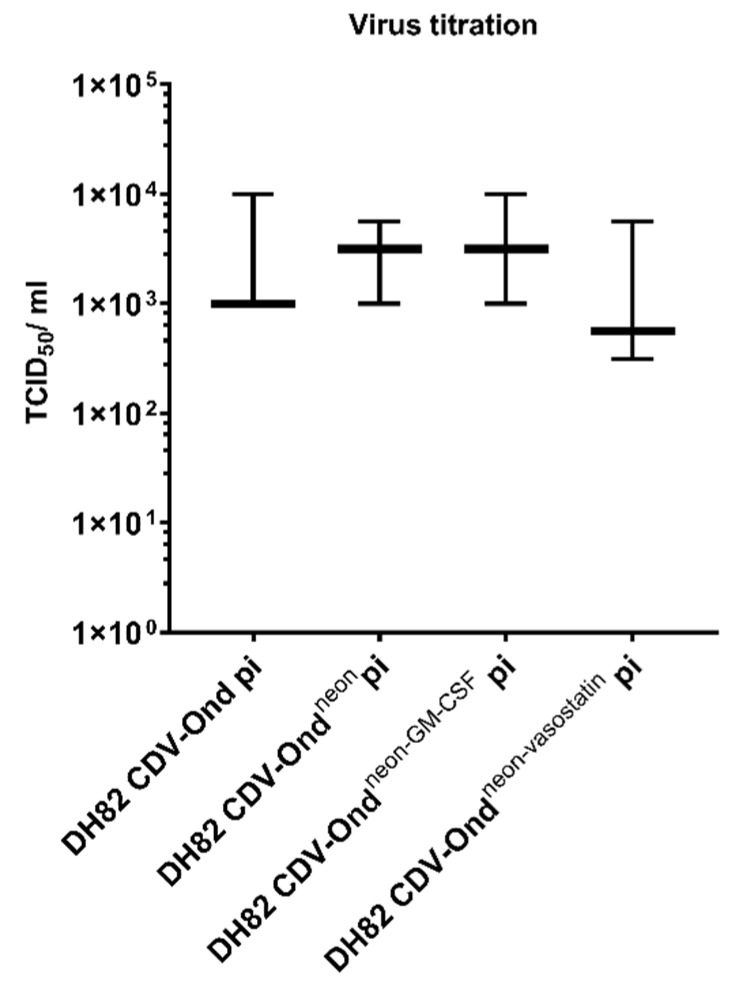
Virus titration revealed no statistically significant differences between the different persistently infected cell lines (Mann–Whitney-U test; *p* > 0.05). Box plots represent minimum, median and maximum.

Virus replication ability was compared using virus titration and revealed no significant differences between the different persistently infected cell lines (*p* > 0.05; Figure 3).

### 2.2. GM-CSF Is Increased in DH82 Cells Persistently Infected with CDV-Ond^neon-GM-CSF^ on the Transcriptional and Protein Level

**Figure 4:**
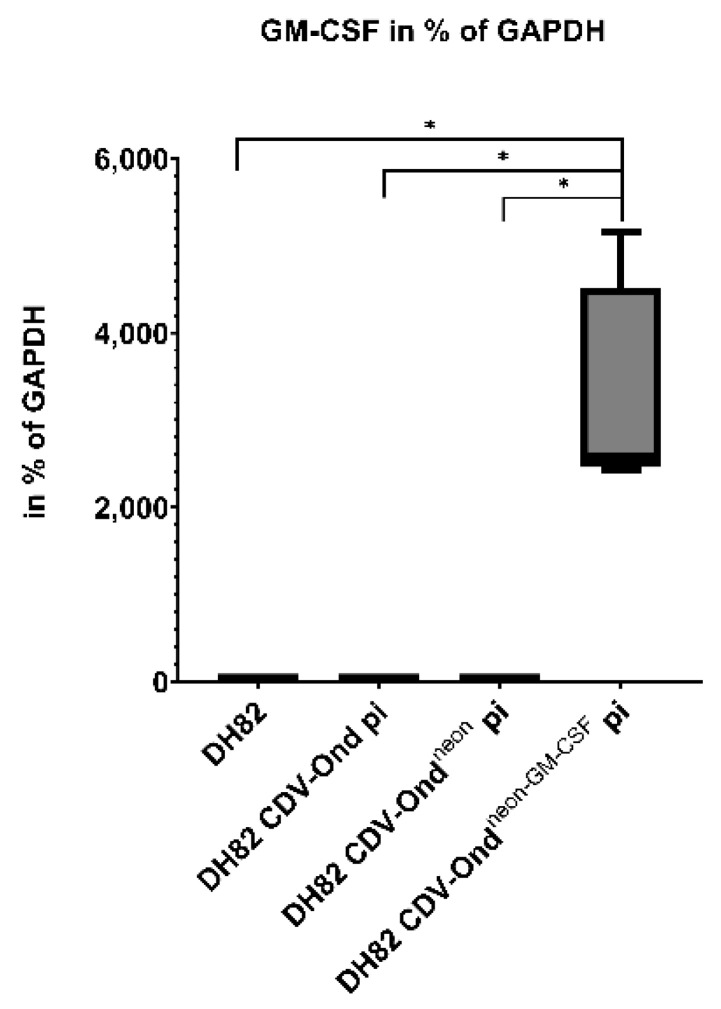
GM-CSF investigation on a transcriptional level in non-infected controls and DH82 cells persistently infected with CDV-Ond, CDV-Ond^neon^ and CDV-Ond^neon-GM-CSF^. The number of GM-CSF mRNA transcripts was significantly higher in DH82 cells persistently infected with CDV-Ond^neon-GM-CSF^ compared to all other cultures. Box plots represent minimum, first quartile, median, third quartile, and maximum. Significant differences (Mann–Whitney-U test) are labeled by asterisks, * = *p* ≤ 0.05.

The number of GM-CSF mRNA transcripts was significantly higher in DH82 CDV-Ond^neon-GM-CSF^ pi cells compared to non-infected controls (*p* = 0.0029), DH82 CDV-Ond pi cells (*p* = 0.0029) and DH82 CDV-Ond^neon^ pi cells (*p* = 0.0029), as shown in Figure 4 and Table 3. 

**Figure 5:**
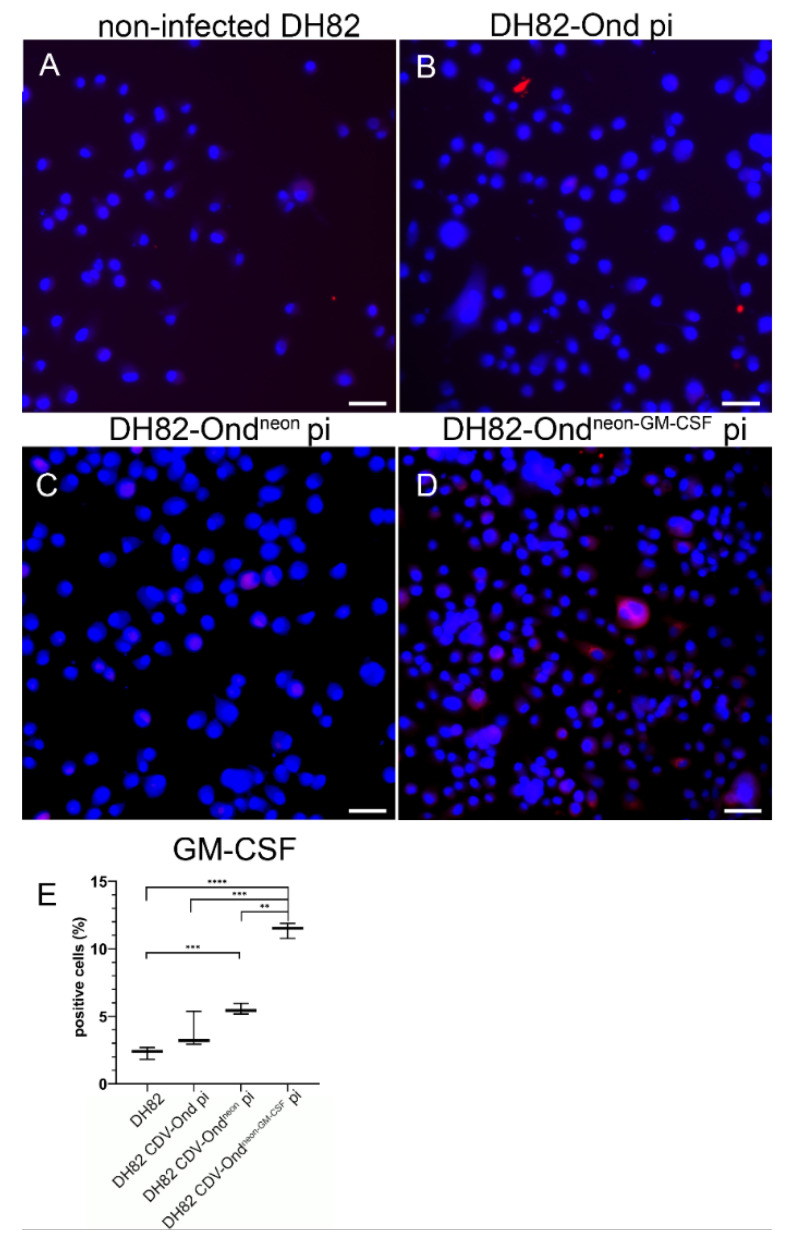
Immunofluorescence using an anti-GM-CSF antibody in non-infected controls and DH82 cells persistently infected with CDV-Ond, CDV-Ond^neon^ and CDV-Ond^neon-GM-CSF^, respectively. Immunostaining of non-infected DH82 cells (**A**) and DH82 cells persistently infected with CDV-Ond (**B**), CDV-Ond^neon^ (**C**), and CDV-Ond^neon-GM-CSF^ (**D**) displayed a significantly higher percentage of positive cells (red) in DH82 cells infected with CDV-Ond^neon-GM-CSF^ compared to all other groups. Nuclei were counterstained with bisbenzimide (blue). Bars = 20 µm. (**E**) Graphical presentation of the percentage of GM-CSF-expressing cells in all analyzed cultures as determined by immunofluorescence. Box plots represent minimum, median and maximum. Significant differences (Mann–Whitney-U test) are labeled by asterisks: ** = *p* < 0.01; *** = *p* < 0.001; **** = *p* < 0.0001.

In order to verify the qPCR data on a protein level, fluorescent immunolabeling was performed. DH82 CDV-Ond^neon-GM-CSF^ pi cells revealed a significantly higher number of GM-CSF immunolabeled cells compared to non-infected DH82 cells (*p* < 0.0001), DH82 CDV-Ond pi cells (*p* = 0.0009) and DH82 CDV-Ond^neon^ pi cells (*p* = 0.0001), respectively, as shown in Figure 5 and Table 4.

**Figure 6:**
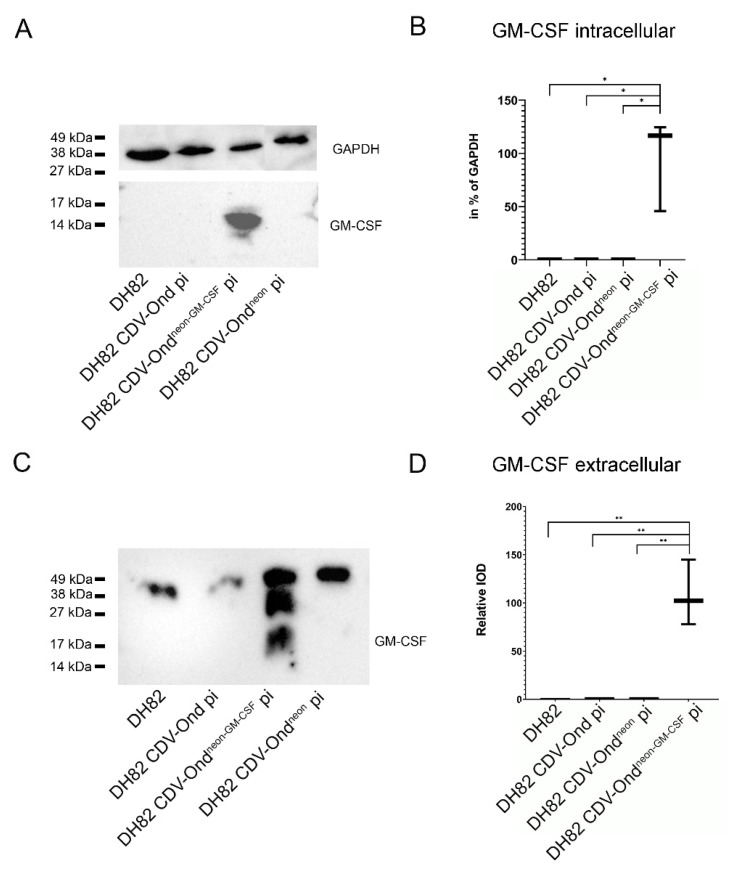
Western blot analysis of the intra- and extracellular amounts of GM-CSF in DH82 cell cultures persistently infected with different CDV strains and non-infected controls. Immunoblotting of intracellular (**A**,**B**) and extracellular (**C**,**D**) proteins with a GM-CSF specific antibody revealed bands at 14 kDa only in DH82 cells infected with CDV-Ond^neon-GM-CSF^ (**A**,**C**). (**B**,**D**) DH82 cells infected with CDV-Ond^neon-GM-CSF^ revealed significantly more GM-CSF both intracellularly (**B**) and within the culture supernatant (**D**) than non-infected controls and DH82 cells persistently infected with CDV-Ond or CDV-Ond^neon^. Box plots represent minimum, median and maximum. Significant differences (Mann–Whitney-U test) are labeled by asterisks: * = *p* ≤ 0.05, ** = *p* < 0.01. IOD—integrated optical density.

Immunofluorescence data were further confirmed by immunoblotting using an anti-canine GM-CSF antibody that showed a band at 14 kDa in the investigated cells. In the densitometrical analysis, DH82 CDV-Ond^neon-GM-CSF^ pi revealed a significantly increased amount of intracellular GM-CSF (*p* = 0.0187) compared to all other groups that did not show any band, as shown in Figure 6. Furthermore, the immunoblotting of culture medium with an anti-canine GM-CSF antibody displayed protein bands with a molecular weight higher than 14 kDa in all groups of persistently CDV-infected DH82 cells and non-infected controls. DH82 CDV-Ond^neon-GM-CSF^ pi cells showed an additional band at 14 kDa. DH82 CDV-Ond^neon-GM-CSF^ pi cells revealed significantly more 14 kDa GM-CSF (*p* = 0.0052) compared to all other groups that did not show any band at 14 kDa (Figure 6).

### 2.3. DH82 Cells Persistently Infected with CDV-Ond^neon-vasostatin^ Show an Increased Number of Vasostatin mRNA Transcripts 

**Figure 7:**
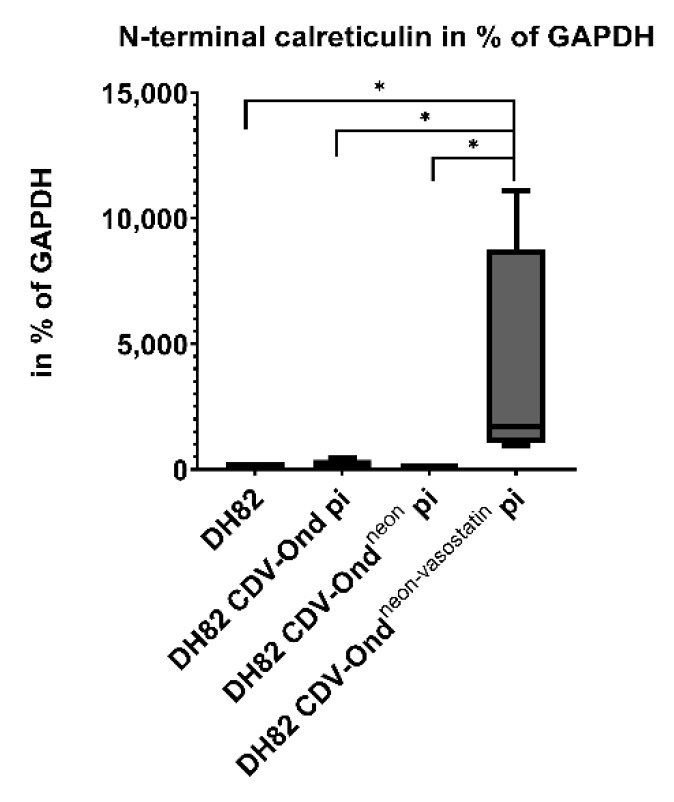
N-terminal calreticulin investigation on a transcriptional level in non-infected DH82 cells and cells persistently infected with CDV-Ond, CDV-Ond^neon^ and CDV-Ond^neon-vasostatin^, respectively. The number of N-terminal calreticulin mRNA transcripts was significantly higher in DH82 cells persistently infected with CDV-Ond^neon-vasostatin^ compared to non-infected controls and DH82 cells persistently infected with CDV-Ond and CDV-Ond^neon^. Box plots represent minimum, first quartile, median, third quartile and maximum. Significant differences (Mann–Whitney-U test) are labeled by asterisks: * = *p* ≤ 0.05.

The number of N-terminal calreticulin (vasostatin) mRNA transcripts was significantly higher in DH82 CDV-Ond^neon-vasostatin^ pi cells compared to non-infected controls (*p* = 0.0286), DH82 CDV-Ond pi (*p* = 0.0286) and DH82 CDV-Ond^neon^ pi cells (*p* = 0.0286), as shown in Figure 7 and Table 5. 

Immunohistochemical staining for N-terminal calreticulin did not display statistically significant differences in the number of immunolabeled cells in all investigated cultures independent of the infection state (Supplementary Figure S4). Immunolabeled cells displayed cytoplasmic to membranous staining (Supplementary Figure S4). 

**Figure 8:**
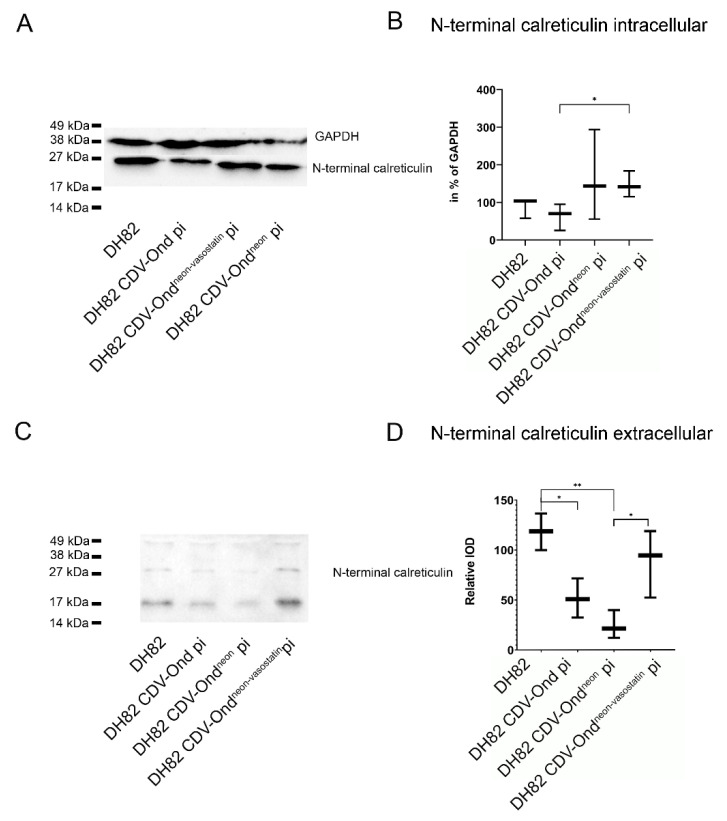
Western blot analysis of intracellular (**A**,**B**) and extracellular (**C**,**D**) proteins using an N-terminal calreticulin antibody. (**A**) Immunoblotting with an N-terminal calreticulin antibody of intracellular proteins showed significant differences in sizes and intensities of bands at 27 kDa between DH82 cells persistently infected with CDV-Ond^neon-vasostatin^ and DH82 cells persistently infected with CDV-Ond. (**B**) Intracellular amount of N-terminal calreticulin in % of GAPDH. Box plots represent minimum, median and maximum. Significant differences (Mann–Whitney U test) are labeled by asterisks: * = *p* ≤ 0.05. (**C**) The supernatant of DH82 cells persistently infected with CDV-Ond^neon-vasostatin^ contained significantly more 27 kDa N-terminal calreticulin compared to DH82 cells persistently infected with CDV-Ond^neon^. In addition, the supernatant of DH82 cells contained significantly more 27 kDa N-terminal calreticulin compared to DH82 cells persistently infected with CDV-Ond and CDV-Ond^neon^. (**D**) Densitometric analysis of the amount of vasostatin within the culture supernatant. Box plots represent minimum, median and maximum. Significant differences (Mann–Whitney U test) are labeled by asterisks: * = *p* ≤ 0.05; ** = *p* < 0.01. IOD—integrated optical density.

The immunoblotting of intracellular proteins with an anti-N-terminal calreticulin antibody revealed detectable bands at 27 kDa, as expected in all investigated cultures. However, densitometrical analysis showed significant differences in sizes and intensities of bands at 27 kDa in DH82 CDV-Ond^neon-vasostatin^ pi cells (median 141.43% of GAPDH; range 115.25–183.87% of GAPDH) compared to DH82 CDV-Ond pi cells (*p* = 0.0432; median 70.21% of GAPDH; range 25.54–95% of GAPDH), as shown in Figure 8. Non-infected DH82 cells and DH82 cells persistently infected with CDV-Ond^neon^ revealed no significant changes compared to DH82 cells persistently infected with CDV-Ond^neon-vasostatin^. Furthermore, the immunoblotting of culture mediums with an anti-N-terminal calreticulin antibody displayed protein bands at 27 kDa in all groups of persistently CDV-infected DH82 cells and non-infected controls. Densitometrical analysis showed that DH82 cells persistently infected with CDV-Ond^neon-vasostatin^ (median 4561; range 3460–7284) expressed significantly more 27 kDa N-terminal calreticulin than cultures persistently infected with CDV-Ond^neon^ (median 3059; range 2141–4348; *p* = 0.044), as shown in Figure 8. Non-infected DH82 cells and DH82 cells persistently infected with CDV-Ond revealed no significant changes compared to DH82 cells persistently infected with CDV-Ond^neon-vasostatin^.

## 3. Discussion

The present study represents an *in vitro* characterization of canine histiocytic sarcoma (DH82) cells persistently infected with canine distemper virus strain Onderstepoort genetically modified to express either vasostatin or granulocyte and macrophage colony-stimulating factor (GM-CSF). Viruses with inserted immunomodulatory or anti-angiogenetic genes broaden the spectrum of mechanisms leading to tumor regression and the prevention of tumor spread and metastases, leading to enhanced oncolytic properties of the armed viruses [28,29,30,31].

The immunolabeling for CDV-nucleoprotein (NP) revealed that up to 98% of DH82 cells were successfully infected with CDV-Ond. Similarly, DH82 cells infected with the genetically engineered viruses (CDV-Ond^neon^ CDV-Ond^neon-vasostatin^ and CDV-Ond^neon-GM-CSF^) revealed a high percentage of infected cells ranging from 86% to 94%. These data show that the genetically engineered viruses behaved similarly to the non-modified CDV-Ond strain regarding the percentage of infected cells. Furthermore, the cumulative population doubling assay revealed that a persistent infection of DH82 cells with the different viruses (CDV-Ond, CDV-Ond^neon^, CDV-Ond^neon-vasostatin^, CDV-Ond^neon-GM-CSF^) had no effect on the growth rate that did not differ significantly over time during the experiment. The present results are in agreement with previously published cumulative population doubling assays of DH82 cells and DH82 cells persistently infected with CDV-Ond [11,13]. 

Measles virus (MV), closely related to CDV, has already been reported to lead to tumor regression in a murine model for human Burkitt’s lymphoma [32]. Interestingly, the insertion of the GM-CSF gene in MV delays murine colon adenocarcinoma progression and prolongs the median overall survival of mice [28]. Interestingly, more than one-third of treated mice recovered completely from neoplasms and rejected tumor re-engraftment, implicating a long-term effect of the treatment with genetically modified MV [28]. The toxicity reports of the cytokine GM-CSF, applied for therapeutical purposes, are promising, with no severe adverse effects after systemic administration in a murine model being found so far [27,28,32]. Since the polarization of M2 (known as anti-inflammatory and pro-tumoral) into M1 macrophages (pro-inflammatory and anti-tumoral) depends mainly on the presence of GM-CSF, this molecule is very attractive in cancer research [20,24,25]. It is interesting to note that canine monocytes have been shown to respond to GM-CSF-induced polarization into M1 in an *in vitro* study [33].

In the present study, DH82 cells persistently infected with CDV-Ond^neon-GM-CSF^ revealed a significantly higher number of GM-CSF mRNA transcripts compared to all other groups. This result suggests that the inserted GM-CSF gene in CDV-Ond^neon-GM-CSF^ is a stable construct in early cell passages. The higher number of GM-CSF transcripts corresponded on the protein level with an increased number of GM-CSF-immunolabeled cells and high amounts of protein, suggesting a CDV-Ond^neon-GM-CSF^ infection-induced effect on DH82 cells. However, the relatively low number of GM-CSF-expressing cells compared to the very high amount of intra- and extra-cellular protein as determined by immunoblotting might originate from single DH82 cells producing very high amounts of this protein after infection with CDV-Ond^neon-GM-CSF^. 

However, Western blot results demonstrated two unexpected protein bands with molecular weights higher than 14 kDa, most likely representing various glycosylated forms of GM-CSF [34]. The low glycosylated form of GM-CSF (14 kDa) has been described as its active form [34]. Nevertheless, future functional tests are needed to confirm the effective functionality of this protein produced by infected DH82 cells. The gene constructs employed in the present study were found to be stable on an mRNA level, as already reported for other negative-strand RNA viruses (ssRNA(-)) [35]. Other CDV-related oncolytic viruses such as Newcastle virus disease (NDV) were successfully modified with a GM-CSF gene insertion exerting antitumoral bystander effects *in vitro* in a tumor neutralization assay via stimulating peripheral blood mononuclear cells [36].

Interestingly, other viruses have also been successfully employed for the same purpose, such as Herpes simplex virus type I with a GM-CSF insertion and deletion of γ34.5 and ICP47 genes, which has been approved by the Food and Drug Administration for advanced melanoma treatment since 2015 [37,38]. One of the leading strategies for the success of GM-CSF therapy is based on the repolarization of macrophages sometimes referred to as re-education [39,40]. In a murine breast cancer allograft model, GM-CSF induced a macrophage re-polarization into the M1 phenotype leading to an inhibition of tumor growth and metastasis [39]. In a previously established canine HS xenograft model using DH82 cells [10,14], high numbers of tumor-associated macrophages (TAMs) were found in acutely CDV-infected xenografts [10] compared to non-infected controls. Interestingly, persistently CDV-infected DH82 xenografts evolved complete spontaneous regression [14], which could not be achieved by ten-fold intratumoral CDV infection alone. *In vitro*, CDV-Ond^neon-GM-CSF^ behaves similarly to the parenteral CDV-Ond strain while producing increased amounts of GM-CSF, rendering the engineered strain highly attractive to enhance the aforementioned mechanism of regression via TAMs. This might be attributed to a higher number of TAMs and additionally by a re-polarization of TAMs into the anti-tumoral M1 phenotype. However, the functionality for an *in vivo* treatment has to be evaluated in further studies. 

In DH82 cells infected with CDV-Ond^neon-vasostatin^, a higher number of vasostatin mRNA transcripts were found. However, the increased level of vasostatin mRNA transcripts was not mirrored on the protein level. Indeed, the number of cells with membranous immunolabeling for vasostatin showed no significant differences between all groups. Unfortunately, the vasostatin was not tagged in the modified CDV-Ond^neon-vasostatin^ strain, rendering a discrimination between virus-induced and regularly cell-produced vasostatin impossible. Moreover, the intracellular and extracellular (culture medium) levels of vasostatin, detected by immunoblotting, further corroborate the fact that there might be a post-translational interference after the vasostatin mRNA translation that prevents the final protein formation. One possible explanation might be the involvement of non-membranous organelles that assemble in response to acute cellular stress, known as stress granules [41] and processing bodies (P-bodies) [42]. They are both reported to induce the inhibition of the translation and storage of biomolecules [41]. One of the causes reported to induce stress granule and P-body formation is cellular stress [42]. Among cellular stressors, viral infection is reported to stimulate particular stress granules’ assembly [41]. This might represent an explanation of the lack of an increased vasostatin protein expression in DH82 CDV-Ond^neon-vasostatin^, despite the high number of vasostatin mRNA transcripts.

The downregulation of calreticulin mRNA levels and expression on the protein level was observed in stress conditions [43]. One of the functions of calreticulin is regulating calcium homeostasis [43]. A decreased amount of calreticulin may be necessary to increase cytosolic Ca^2+^ levels under stress conditions [43]. Cellular stress is associated with calreticulin fluctuation, which may be an explanation for the unexpected Western blot results in the present study. Minor bands seen in SDS-PAGE electrophoresis may be artefacts of proteolysis [44]. Moreover, it is also reported that calreticulin may form aggregates [44]. These properties of investigated protein may result in an observed, unexpected higher molecular weight of the products. Future studies are needed in order to better understand the cause underlying the lack of increased vasostatin production on a protein level in DH82 CDV-Ond^neon-vasostatin^ cells and to verify its functionality. 

In conclusion, the *in vitro* characterization on a transcriptional and protein level of two CDV-Ond strains engineered for expressing vasostatin and GM-CSF, respectively, revealed that CDV-Ond, as a ssRNA(-) virus [35], is a good candidate for successful gene construct insertions. Increased vasostatin mRNA transcripts render CDV-Ond^neon-vasostatin^ a good candidate for future functional studies *in vitro* and *in vivo*. The production and secretion of GM-CSF by DH82 cells persistently infected with CDV-Ond^neon-GM-CSF^ confirmed this candidate as a promising tool for future functional *in vitro* studies and *in vivo* murine xenograft investigations in a model for HS using DH82 cells. 

The current study focused on early passages of persistently CDV-infected DH82 cells to maximize potential effects due to very high percentages of CDV-positive cells. Future studies should also focus on higher passages in order to demonstrate that the virus is able to constantly infect the cell population and to see if this will affect the production of vasostatin or GM-CSF.

## 4. Materials and Methods

### 4.1. Cell Culture

Non-infected canine histiocytic sarcoma (DH82) cells obtained from the European Collection of Authenticated Cell Cultures (ECACC No. 94062922) and persistently canine distemper virus (CDV) strain Onderstepoort (CDV-Ond)-infected DH82 cells (DH82 CDV-Ond pi) were cultured as previously described [11,12,13,14]. Briefly, cells were maintained in Minimal Essential Medium (MEM) with Earle’s salts (Merck, Darmstadt, Germany) supplemented with 10% fetal bovine serum (Capricorn Scientific, Ebsdorfergrund, Germany), 1% penicillin/streptomycin (Sigma-Aldrich, Taufkirchen, Germany) and 1% non-essential amino acids (Sigma-Aldrich, Taufkirchen, Germany). DH82 CDV-Ond pi were obtained as formerly described [9]. Passage 45 of the DH82 cells was infected with CDV-Ond^neon^, CDV-Ond^neon-vasostatin^ or CDV-Ond^neon-GM-SCF^ with 100 µL of the stock solution. After inoculation for 2 h in the incubator, the inoculum was replaced by culture medium. After reaching a persistent infection state of DH82 cells infected with CDV-Ond^neon^, CDV-Ond^neon-vasostatin^ and CDV-Ond^neon-GM-CSF^, cells were passaged every 7–10 days when the cultures were nearly 90% confluent. All cells were cultured in T75 (ThermoFischer Scientific, Schwerte, Germany) and T25 flasks (ThermoFischer Scientific, Schwerte, Germany) in an incubator at standard conditions (37 °C, 5% CO_2_ in a water saturated atmosphere). The medium was changed every 2–3 days. In the present study, experiments were performed using passage 47 of the non-infected DH82 cells and passage 172 of the persistently CDV-Ond-infected DH82 cells. Moreover, due to a decreased number of cells expressing mNeonGreen in the later passages, passages 4–6 of DH82 cells persistently infected with CDV-Ond^neon^, CDV-Ond^neon-GM-CSF^ or CDV-Ond^neon-vasostatin^ were used. 

### 4.2. Cumulative Population Doubling Assay

To investigate cell growth rate, a cumulative population doubling assay was performed. Non-infected DH82 cells and DH82 CDV-Ond pi, DH82 CDV-Ond^neon^ pi, DH82 CDV-Ond^neon-vasostatin^ pi and DH82 CDV-Ond^neon-GM-CSF^ pi cells were seeded into T75 flasks and cultured over 3 weeks. The medium was changed every 2–3 days and the cells were counted at every cell passage performed in weekly intervals. The population doubling (PD) was calculated as previously described [11,13] according to the following formula: PD = log_10_ (cells harvested- initial cell number)/log_2_ [45]. The cumulative population doubling was performed by adding the PD of each passage to the PD of the previous one. 

### 4.3. Genetically Engineered Virus Strains

**Figure 9:**
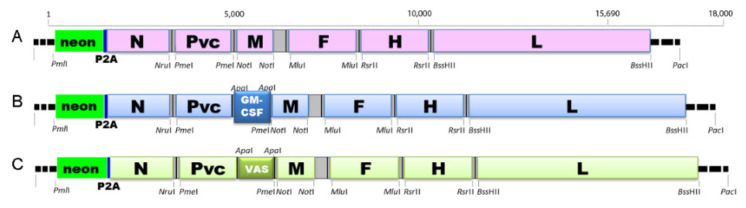
Constructs of the modified viruses: DH82 CDV-Ond^neon^ (**A**), CDV-Ond^neon-GM-CSF^ (**B**) and DH82 CDV-Ond^neon-vasostatin^ (**C**).

Recombinant viruses used in this study were derived from CDV-Ond^neon^, as previously described [46,47]. Subsequently, viruses were further engineered to express either canine GM-CSF or vasostatin. To this aim, an additional transcription cassette (including the foreign gene canine GM-CSF (CSF2_CANLF) or canine vasostatin (N-terminal fragment (1–180 aa) of the canine calreticulin (F6UYJ9_CANLF)) flanked by the gene start and stop signals of the N gene) was inserted in between the P and M genes (Figure 9).

### 4.4. Virus Titration

Virus titration was performed as previously described [48]. Briefly, the supernatants of non-infected DH82 cells and DH82 cells persistently infected with CDV-Ond^neon^, CDV-Ond^neon-vasostatin^ and CDV-Ond^neon-GM-CSF^ were centrifuged (700× *g*, 10 min, 4 °C) and diluted logarithmically from 10^0^ to 10^−10^ in Dulbecco’s Modified Eagle’s Medium (Merck, Darmstadt, Germany) containing 10% fetal bovine serum (Capricorn Scientific, Ebsdorfergrund, Germany), 1% penicillin/streptomycin (Sigma-Aldrich, Taufkirchen, Germany) and zeocin (InvivoGen, Toulouse, France) and titrated in quadruplicates in 96-well microtiter plates (ThermoFischer Scientific, Schwerte, Germany) containing Vero.DogSLAM cells (1.5 × 10^4^/well). Following the 7-day incubation period under standard conditions, the cells were examined for cytopathogenic effect. In addition, the 50% log_10_ tissue culture infectious dose per milliliter (TCID_50_/mL) was calculated. Analysis was performed in three independent samples per group.

### 4.5. RNA Isolation and cDNA Synthesis 

The total RNA of non-infected DH82 cells and DH82 CDV-Ond pi, DH82 CDV-Ond^neon^ pi, DH82 CDV-Ond^neon-vasostatin^ pi and DH82 CDV-Ond^neon-GM-CSF^ pi cells was extracted using TRIzol (Invitrogen, Carlsbad, CA, USA) and purified with RNeasy Mini Kit (Qiagen, Hilden, Germany) and RNase free DNase Kit (Qiagen, Hilden, Germany) according to the manufacturer’s protocols. The RNA concentration was spectroscopically measured at 260 nm using the GeneQuant pro (GE Healthcare, Amersham, Buckinghamshire, United Kingdom). Transcription to cDNA was performed using Omniscript (Qiagen, Hilden, Germany), RNAse OUT (ThermoFischer Scientific, Schwerte, Germany) and Random Hexamers (Promega, Madison, WI, USA) according to the manufacturer’s protocols. Reverse transcription was performed using a Biometra Thermocycler T-Gradient ThermoBlock (American Laboratory Trading, East Lyme, CT, USA) under the following conditions: 25 °C for 10 min, 37 °C for 1 h and 93 °C for 5 min. 

### 4.6. Primer Design

Primers were designed using Primer3 web version 4.1.0 or taken from the literature [9,49]. All primers are listed in Table 6.

### 4.7. Qualitative PCR 

Qualitative PCR was performed using a Biometra Thermocycler T-Gradient ThermoBlock (American Laboratory Trading, East Lyme, CT, USA) under the following conditions: denaturation at 94 °C for 1 min followed by 40 cycles of denaturation at 94 °C for 1 min, annealing at 55 °C (vasostatin), 58 °C (GAPDH, GM-CSF) or 59 °C (CDV) for 2 min, extension at 72 °C for 1 min and a final extension at 72 °C for 5 min using the Taq DNA Polymerase Kit (Invitrogen, Carlsbad, CA, USA) with 1.25 mM MgCl_2_, 0.2 mM dNTPs Mix (New England Biolabs, Ipswich, MA, USA) and primers at a concentration of 300 nM each. Gel electrophoresis for PCR amplicons was run in 2% agarose gel (Biozym LE Agarose, Hessisch Oldendorf, Germany) over 45 min at 90 V, 100 W and 150 mA. 

### 4.8. Reverse Transcription Quantitative RT-qPCR

Standard curves for estimation of copy numbers were generated using RT-PCR amplicons from 10^2^ to 10^8^ copies per sample. Reverse transcription quantitative PCRs were performed in quadruplicates with negative controls as previously described [9]. The NucleoSpin^®^ Gel and PCR Clean-up Kit (Macherey-Nagel, Düren, Germany) was used for isolation of PCR amplicons out of the agarose gel according to the manufacturer’s protocol. Standard curves were prepared using serial-diluted PCR amplicons in a concentration ranging from 10^2^ to 10^8^ copies/µL. Quantitative PCR was performed using the Brilliant III Ultra-Fast SYBR^®^ Green QPCR Master Mix (Agilent Technologies, Santa Clara, CA, USA). Primers were diluted to a final concentration of 150 nM each. The reaction was performed using the AriaMx Real-time PCR System (Agilent Technologies, Santa Clara, CA, USA) at the following conditions: denaturation at 95° for 3 min and 40 cycles at 95 °C for 5 s, 57 °C (CDV), 58 °C (GM-CSF), 60 °C (vasostatin) or 64 °C (GAPDH) for 10 s, followed by one cycle at 95 °C for 30 s, 65 °C for 30 s and 95 °C for 30 s. The melting curve was established by initial denaturation at 95 °C for 1 min, followed by 40 cycles, starting with 55 °C and increasing the temperature by 1 °C per cycle. GAPDH was used as a housekeeping gene for standardization of relative gene expression. The relative gene expression was calculated with the formula X/Y × 100, where X = gene expression of the target and Y = expression level of the housekeeping gene (GAPDH). 

### 4.9. Immunoblotting

Immunoblotting of non-infected DH82 and DH82 CDV-Ond pi, DH82 CDV-Ond^neon^ pi, DH82 CDV-Ond^neon-vasostatin^ pi and DH82 CDV-Ond^neon-GM-CSF^ pi was carried out in three independent samples from each cell type as formerly described [11,12,50]. For cell lysis, RIPA buffer (150 mM NaCl, 1% Triton X, 0.5% sodium deoxycholate, 0.1% SDS, 50 mM Tris) with the addition of the Protease Inhibitor Cocktail (Sigma-Aldrich, Taufkirchen, Germany) was used. The concentration of proteins was determined using the Pierce^TM^ BCA Protein Assay Kit (Thermo Scientific, Schwerte, Germany). For protein normalization, glyceraldehyde 3-phosphate dehydrogenase (GAPDH) was used as a housekeeping protein. The same amount of proteins were separated on 15% SDS-PAGE gels. Subsequently, proteins were transferred to a nitrocellulose membrane (Bio-Rad, Hercules, CA, USA) blocked with 5% milk (Merck, Darmstadt, Germany) in 0.1% Tween 20 in phosphate-buffered saline (PBS) for 1 h. Immunoblotting was performed using a primary antibody directed against canine GM-CSF (1:1000; R&D Systems, Minneapolis, MN, USA), N-terminal calreticulin (1:1000; Sigma-Aldrich, Taufkirchen, Germany) and GAPDH (1:1000; Sigma-Aldrich, Taufkirchen, Germany). Horseradish peroxidase (HRP)-conjugated rabbit anti goat (1:1000; R&D Systems; Minneapolis, MN, USA), goat anti rabbit (1:1000; Thermo Scientific, Schwerte, Germany) and rabbit anti mouse (1:1000; Invitrogen, CA, USA) antibodies were used as secondary antibodies. Protein bands were visualized using chemiluminescent substrate (SuperSignal^TM^ West Pico PLUS, Thermo Scienific, Schwerte, Germany) and a ChemiDoc MP Imaging System (Bio-Rad, Hercules, CA, USA). Densitometric analysis was performed to quantify band sizes and intensities using ImageJ version 1.51.0 (https://imagej.nih.gov/ij/). Subsequently, intracellular GM-CSF and N-terminal calreticulin amounts were normalized against the amount of GAPDH.

### 4.10. Immunohistochemistry

To investigate the cellular localization of vasostatin, immunohistochemistry for N-terminal calreticulin was performed. Formalin-fixed paraffin-embedded (FFPE) non-infected DH82, DH82 CDV-Ond pi, DH82 CDV-Ond^neon^ pi and DH82 CDV-Ond^neon-vasostatin^ pi cell pellets were obtained. Two to three-micrometer-thick slides were obtained from each cell pellet. Immunolabeling was performed in triplicates with negative controls as previously described [11,51,52]. Briefly, after dewaxing, rehydration and blocking of endogenous peroxidases, sections were exposed to antigen retrieval with citrate buffer. After serum blocking for 30 min, slides were incubated overnight at 4 °C with the primary antibody. Afterwards, a secondary biotinylated antibody and an avidin–biotin complex (ABC) peroxidase kit (Vectastain^®^ Elite^®^ ABC Kit, Vector Laboratories, Burlingame, CA, USA) were applied for 30 min and 20 min, respectively. A 3′3′-diaminobenzidine (DAB) system (Vector Laboratories, Burlingame, CA, USA) was used for the detection of positive reactions. Nuclei were counterstained with Mayer’s hemalum (Carl Roth GmbH, Karlsruhe, Germany). Information regarding antibody details, antigen retrieval and blocking serum are reported in Table 7. For negative controls, the specific primary antibody was replaced by ascitic fluid from non-immunized BALB/cJ mice. The dilution of the negative controls was chosen according to the protein concentration of the replaced primary antibodies. The percentage of immunopositive cells was assessed manually for non-infected DH82 cells, DH82 CDV-Ond pi, DH82 CDV-Ond^neon^ pi, DH82 CDV-Ond^neon-vasostatin^ pi and DH82 CDV-Ond^neon-GM-CSF^ pi. Therefore, pictures were taken in 5 evenly distributed fields per cell pellet at a 200× magnification using a microscope (Olympus BX51, Olympus optical Co. GmbH, Hamburg, Germany) equipped with an Olympus D72 camera (Olympus optical Co. GmbH, Hamburg, Germany). For analysis, the relative number of cells with membranous staining was counted and divided by the total number of cells.

### 4.11. Immunofluorescence

In order to verify the GM-CSF production within the investigated cells and the infection status with CDV-Ond, the cells were immunolabeled for anti-canine GM-CSF and CDV-nucleoprotein (NP), respectively. The immunostaining of DH82 cells was performed in triplicates with negative controls in duplicates in 96-well microtiter plates (ThermoFischer Scientific, Schwerte, Germany), as previously described [11]. Briefly, cells were seeded at a density of 20,000 cells/well, fixed with 4% buffered paraformaldehyde (PFA 4%, pH 7.4) and permeabilized with PBS-Triton X (0.025%). After serum blocking, cells were incubated overnight at 4 °C with the primary antibody. Afterwards, cells were washed with PBS/0.1% Triton and incubated for 2 h with the secondary antibody. For nuclear staining, bisbenzimide (Merck, Darmstadt, Germany) was used. Antibody details, antigen retrieval and blocking serum are reported in Table 7. Negative controls included the omission of primary or secondary antibodies, respectively. The percentage of immune-positive cells was assessed manually for non-infected DH82 cells, DH82 CDV-Ond pi, DH82 CDV-Ond^neon^ pi, DH82 CDV-Ond^neon-vasostatin^ pi and DH82 CDV-Ond^neon-GM-CSF^ pi in 5 randomly selected fields per well. Therefore, pictures were taken at a 200× magnification using a microscope (Olympus IX-70, Olympus optical Co. GmbH, Hamburg, Germany) equipped with an Olympus DP-72 camera and using Olympus CellSense standard software version 2.3. For analysis, the number of positive cells was counted and divided by the total number of cells.

### 4.12. Statistical Analysis 

For descriptive statistics, median and range were calculated. For the analysis of data obtained from the cell population doubling assay, immunofluorescence, immunoblotting, virus titration, immunohistochemistry and RT-qPCR, the non-parametric Mann–Whitney U test was used. Statistical analysis of mNeonGreen and CDV-NP expression was performed using the Wilcoxon signed-rank test. Statistical analysis was performed with SAS software version 7.1.5.0 (SAS Institute, Cary, NC, USA, www.sas.com). The level of significance was set at *p* ≤ 0.05. Graph creation was carried out using GraphPadPrism version 8.0.1 for Windows (GraphPad Software, La Jolla, CA, USA, www.graphpad.com). 

## Figures and Tables

**Table 1:** Median and range of the number of CDV-Ond-infected cells assessed by CDV nucleoprotein (D110) immunofluorescence.

Percentage of CDV-Infected Cells	Median (in %)	Range (in %)
Non-infected DH82	0	0
DH82 CDV-Ond pi	98.26	97.67–99.12
DH82 CDV-Ond^neon^ pi	96.76	96.08–97.13
DH82 CDV-Ond^neon-vasostatin^ pi	95.56	95.44–97.91
DH82 CDV-Ond^neon-GM-CSF^ pi	97.88	97.30–98.00

DH82—canine histiocytic sarcoma cell line; Ond—canine distemper virus strain Onderstepoort; pi—persistently infected; GM-CSF—granulocyte and macrophage colony-stimulating factor.

**Table 2:** Median and range of the number of mNeonGreen-expressing cells.

Percentage of mNeonGreen-Expressing Cells	Median (in %)	Range (in %)
Non-infected DH82	0	0
DH82 CDV-Ond pi	0	0
DH82 CDV-Ond^neon^ pi	92.19	91.89–92.53
DH82 CDV-Ond^neon-vasostatin^ pi	88.44	83.60–93.34
DH82 CDV-Ond^neon-GM-CSF^ pi	90.63	89.32–94.90

DH82—canine histiocytic sarcoma cell line; CDV-Ond—canine distemper virus strain Onderstepoort; pi—persistently infected; GM-CSF—granulocyte and macrophage colony-stimulating factor.

**Table 3:** Median and range of the number of GM-CSF mRNA transcripts in % of GAPDH.

Number of GM-CSF mRNA Transcripts	Median (in % of GAPDH)	Range (in % of GAPDH)
Non-infected DH82	0.09	0.07–0.14
DH82 CDV-Ond pi	0.08	0.00–0.29
DH82 CDV-Ond^neon^ pi	0.11	0.02–0.26
DH82 CDV-Ond^neon-GM-CSF^ pi	3183.90	2422.74–5158.06

DH82—canine histiocytic sarcoma cell line; CDV-Ond—canine distemper virus strain Onderstepoort; pi—persistently infected; GM-CSF—granulocyte and macrophage colony-stimulating factor.

**Table 4:** Median and range of cells expressing GM-CSF as determined by immunofluorescence.

Percentage of GM-CSF Expressing Cells	Median (in %)	Range (in %)
Non-infected DH82	2.41	1.82–2.70
DH82 CDV-Ond pi	3.20	2.93–5.38
DH82 CDV-Ond^neon^ pi	5.43	5.18–5.43
DH82 CDV-Ond^neon-GM-CSF^ pi	11.53	10.77–11.89

DH82—canine histiocytic sarcoma cell line; CDV-Ond—canine distemper virus strain Onderstepoort; pi–persistently infected; GM-CSF–granulocyte and macrophage colony-stimulating factor.

**Table 5:** Median and range of the number of N-terminal calreticulin (vasostatin) mRNA transcripts in % of GAPDH.

Vasostatin mRNA Transcripts (in % of GAPDH)	Median (in %)	Range (in %)
Non-infected DH82	87.36	56.28–160.97
DH82 CDV-Ond pi	96.33	11.92–472
DH82 CDV-Ond^neon^ pi	36.22	17.55–137.98
DH82 CDV-Ond^neon-vasostatin^ pi	1688.40	923.58–11,081.08

DH82—canine histiocytic sarcoma cell line; CDV-Ond—canine distemper virus strain Onderstepoort; pi—persistently infected.

**Table 6:** Summary of primers used for qualitative and quantitative RT-PCR, expected amplicon length and GenBank accession number.

Genes	Primer Sequences (5′-3′)		Length of Amplicon (bp)	Position	GenBank Accession Number
**GAPDH**	Forward *	AAGGTCGGAGTCAACGGATT	365	7–26	AB038240 [9]
	Reverse *	GCAGAAGAAGCAGAGATGATG		371–351	
	Forward	GTCATCAACGGGAAGTCCATCTC	84	96–218	
	Reverse	AACATACTCAGCACCAGCATCAC		279–257	
**CDV**	Forward *	ACAGGATTGCTGAGGACCTAT	287	769–789	AF378705 [9,49]
	Reverse *	CAAGATAACCATGTACGGTGC		1055–1035	
	Forward	GCTCTTGGGTTGCATGAGTT	83	954–973	
	Reverse	GCTGTTTCACCCATCTGTTG		1036–1017	
**Vasostatin**	Forward *	TGTTCTCAGTTCCGGCAAGT	235	83–103	XM038428450.1
	Reverse *	ACATGCACGGAGACTCTGAG		317–298	
	Forward	TGACCAGGAGAAGGATAAAGGG	100	110–132	
	Reverse	GCAACAAAGGCCAGACACTG		209–189	
**GM-CSF**	Forward *	CTCACCCACCCTTGTCACTC	218	63–83	NM001003245.1
	Reverse *	CACCAGCCTCAAGAATCCCT		280–260	
	Forward	TTGTCACTCGGCCCTCTCA	93	74–93	
	Reverse	TCATCACAGCAGTCACGTCA		160–140	

CDV—canine distemper virus; GAPDH—glyceraldehyde 3-phosphate dehydrogenase; GM-CSF—granulocyte and macrophage colony-stimulating factor; *—primers used for qualitative RT-PCR.

**Table 7:** Summary of antibodies used for immunostaining including primary antibodies, host species, clonality, blocking serum, dilution and secondary antibodies.

Primary Antibody	Host Species, Clonality	Antigen Retrieval	Blocking Serum	Dilution	Secondary Antibody (1:200)
CDV-NP (University of Bern, Prof. Zurbriggen)	Mouse, monoclonal, clone D110	na	Goat serum	1:100 (IF)	GaM-Cy3
GM-CSF (R&D Systems, Minneapolis, MN, USA)	Goat, polyclonal	na	Horse serum	1:80 (IF)	DaG-Cy3
N-terminal calreticulin (Sigma-Aldrich, Taufkirchen, Germany)	Rabbit, polyclonal	Citrate buffer, MW (800 W, 20′)	Goat serum	1:100 (IHC)	GaR-b

CDV-NP—canine distemper virus nucleoprotein; DaG-Cy3—donkey anti goat cyanine 3-conjugated; GM-CSF—granulocyte and macrophage colony-stimulating factor; GaM-Cy3—goat anti mouse cyanine 3-conjugated; GaR-b—goat anti rabbit biotinylated; IF—immunofluorescence; IHC—immunohistochemistry; MW—microwave; na—not applicable.

## Data Availability

All relevant data are included in the manuscript, supplementary material or can be obtained from the authors on reasonable request.

## Supplementary Materials

The following data and information are available online at https://www.mdpi.com/article/10.3390/ijms23116156/s1.
