# Supplementary material for: Persistent Infection of a Canine Histiocytic Sarcoma Cell Line with Attenuated Canine Distemper Virus Expressing Vasostatin or Granulocyte-Macrophage Colony-Stimulating Factor
Source: Int J Mol Sci. 2022 May 31;23(11):6156. doi: 10.3390/ijms23116156 (PMC9181094; doi:10.3390/ijms23116156)
Supplement: Supplementary file 1 [file ijms-23-06156-s001.zip › ijms-1704896-supplementary.pdf]

Supplementary material

Persistent Infection of a Canine Histiocytic Sarcoma Cell Line with Attenuated Canine Distemper Virus Expressing Vasostatin or Granulocyte-Macrophage Colony-Stimulating Factor

Katarzyna Marek [1,2,†], Federico Armando [1,†], Vanessa Maria Nippold [1], Karl Rohn [3], Philippe Plattet [4], Graham Brogden [5,6,7], Gisa Gerold [5,6,8,9], Wolfgang Baumgärtner [1,2,*] and Christina Puff [1]

[1] Department of Pathology, University of Veterinary Medicine Hannover, 30559 Hannover, Germany; katarzyna.marek@tiho-hannover.de (K.M.); federico.armando@tiho-hannover.de (F.A.); pfankuche@laboklin.com (V.M.N.); christina.puff@tiho-hannover.de (C.P.)
[2] Center for Systems Neuroscience, 30559 Hannover, Germany
[3] Institute for Biometry, Epidemiology and Information Processing, University of Veterinary Medicine Hannover, 30559 Hannover, Germany; karl.rohn@tiho-hannover.de
[4] Division of Experimental Clinical Research, Vetsuisse University Bern, 3012 Bern, Switzerland; philippe.plattet@vetsuisse.unibe.ch
[5] Department of Biochemistry, University of Veterinary Medicine Hannover, 30559 Hannover, Germany; graham.brogden@tiho-hannover.de (G.B.); gisa.gerold@tiho-hannover.de (G.G.)
[6] Research Center for Emerging Infections and Zoonoses (RIZ), University of Veterinary Medicine Hannover, 30559 Hannover, Germany;
[7] Institute for Experimental Virology, TWINCORE, Centre for Experimental and Clinical Infection Research, A Joint Venture Between the Medical School Hannover and the Helmholtz Centre for Infection Research, 30625 Hannover, Germany
[8] Wallenberg Centre for Molecular Medicine (WCMM), Umeå University, 901 87 Umeå, Sweden
[9] Department of Clinical Microbiology, Virology, Umeå University, 901 87 Umeå, Sweden
* Correspondence: wolfgang.baumgaertner@tiho-hannover.de
† These authors contributed equally to this work.

Supplementary material:

This file includes:

Supplementary figures S1-S4

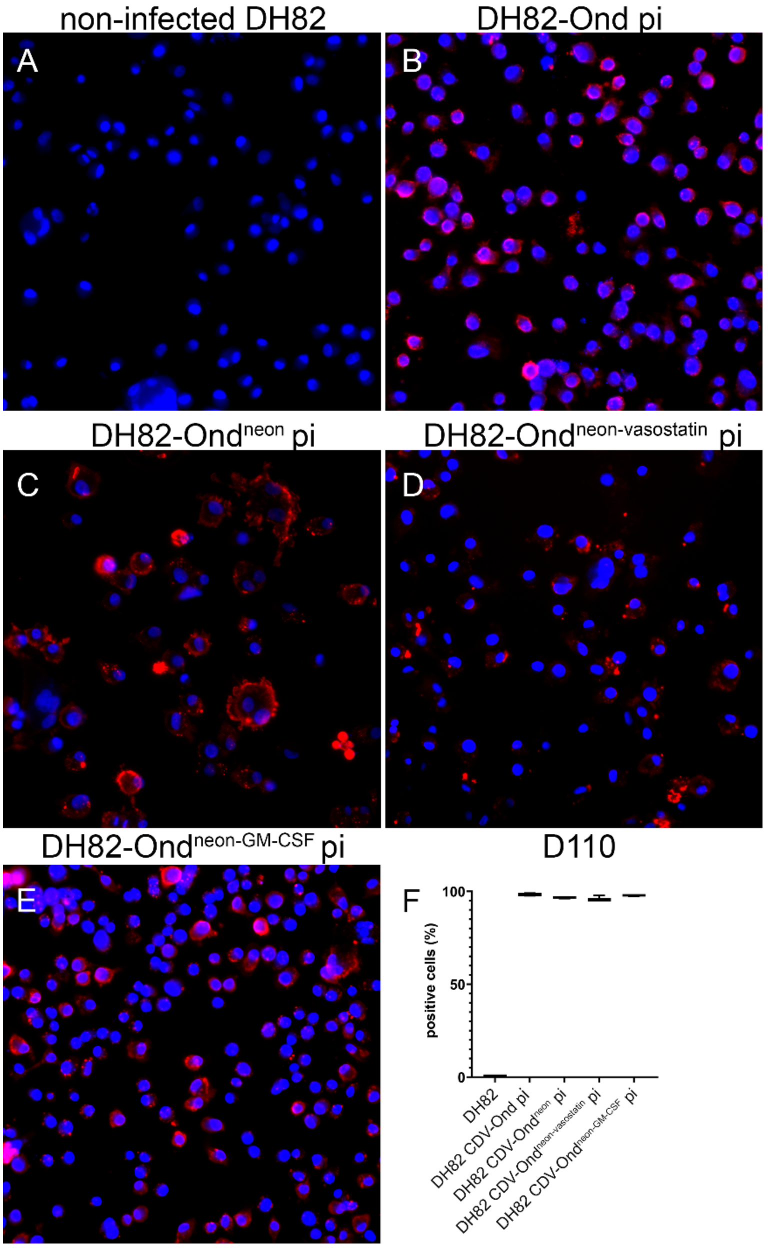

Supplementary Figure S1. (A) Immunofluorescence revealed that non-infected DH82 cells were negative for CDV-nucleoprotein. DH82 CDV-Ond pi (B), DH82 CDV-Ond^{neon} pi (C), DH82 CDV-Ond$^{neon\text{-}vasostatin}$ (D) and DH82 CDV-Ond$^{neon\text{-}GM\text{-}CSF}$ (E) cells were more than 95% positive for CDV-nucleoprotein. Nuclei were labeled with bisbenzimide (blue). Bar = 20µm. (F) Box plots represent minimum, median and maximum.

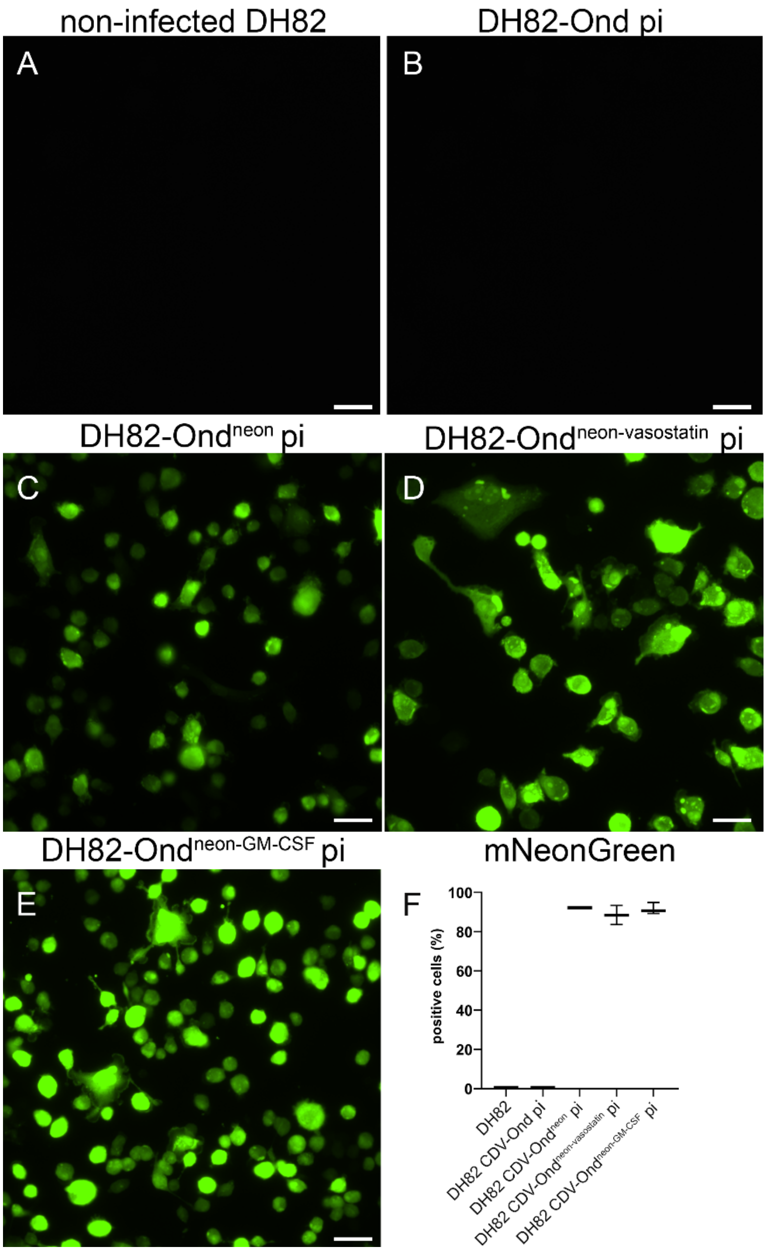

Supplementary Figure S2. Determination of mNeonGreen positive cells revealed that non-infected controls (A) and DH82 cells infected with CDV-Ond (B) were negative. In DH82 CDV-Ond^{neon} pi (C), DH82 CDV-Ond$^{neon-vasostatin}$ (D) and DH82 CDV-Ond$^{neon-GM-CSF}$ (E) cultures more than 80% of the cells expressed mNeonGreen. Bar = 20µm. (F) Box plots represent minimum, median and maximum.

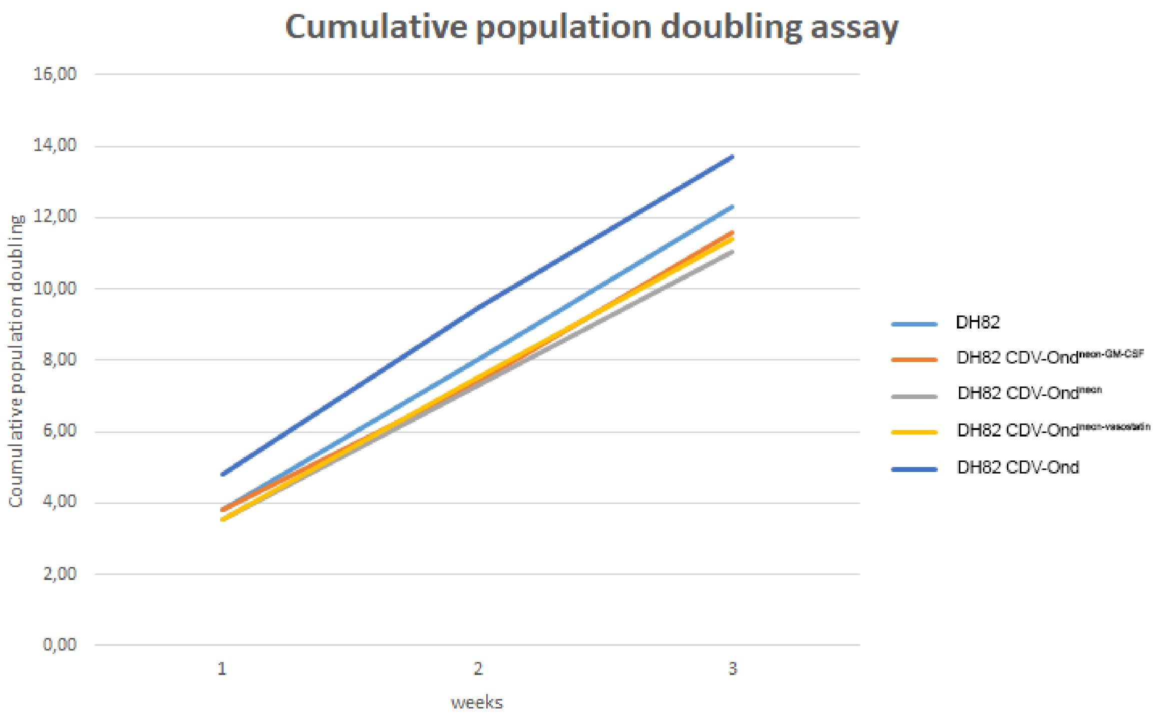

Supplementary Figure S3. Cumulative population cell doubling (CPD) assay revealed that all cultures independent of the virus strain showed a similar growth rate.

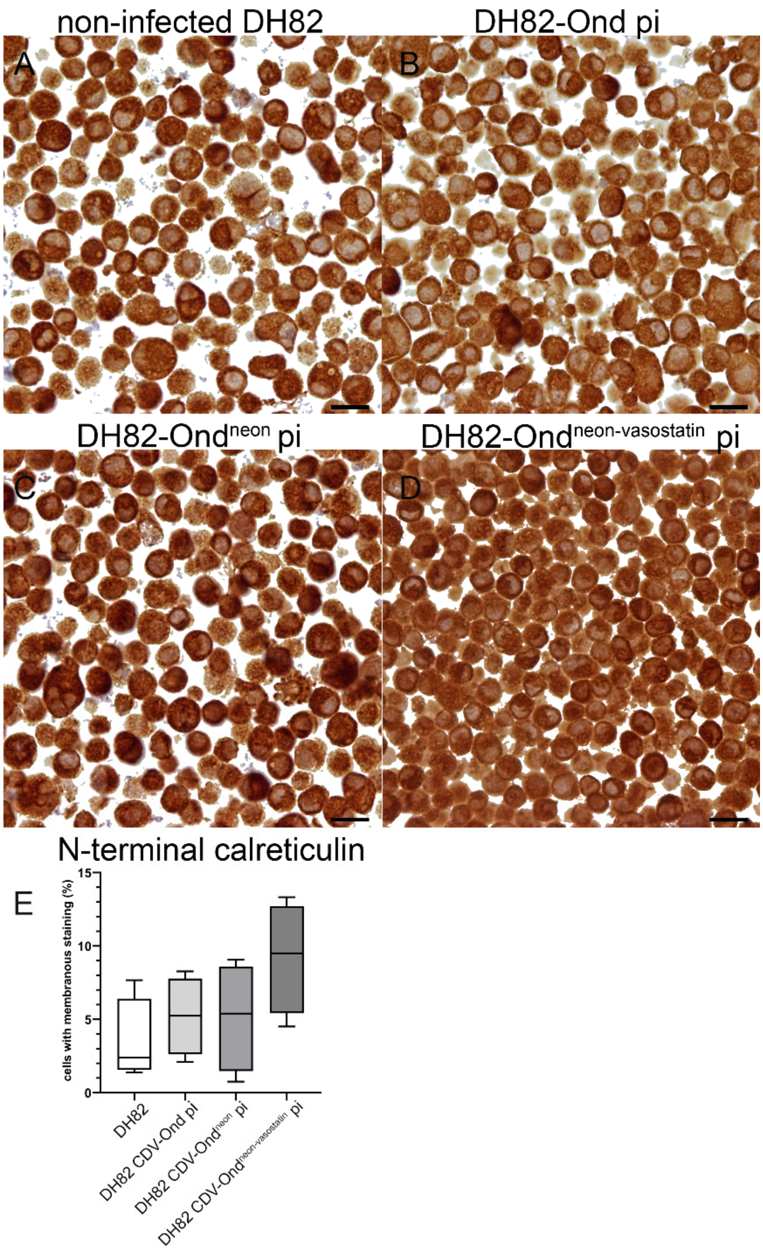

Supplementary Figure S4. Immunohistochemistry for N-terminal calreticulin of non-infected DH82 cells (A) and DH82 cells persistently infected with CDV-Ond (B), CDV-Ond^{neon} (C) and CDV-Ond$^{neon-vasostatin}$ (D) displayed no significant differences in the intracellular protein distribution ($p \geq 0.05$, Mann-Whitney-U test). Bar = 20µm. (E) Graphical presentation of the percentage of cells with a membranous expression of N-terminal calreticulin in non-infected controls and DH82 cells infected with CDV-Ond, CDV-Ond^{neon} and CDV-Ond$^{neon-vasostatin}$. Box plots represent minimum, first quartile, median, third quartile and maximum.
